# A gaming app developed for vestibular rehabilitation improves the accuracy of performance and engagement with exercises

**DOI:** 10.3389/fmed.2023.1269874

**Published:** 2023-11-24

**Authors:** Linda J. D’Silva, Tarah Phongsavath, Kelly Partington, Nathan T. Pickle, Katherine Marschner, Timothy P. Zehnbauer, Michael Rossi, Karen Skop, Paulien E. Roos

**Affiliations:** ^1^Department of Physical Therapy, Rehabilitation Science, and Athletic Training, University of Kansas Medical Center, Kansas, MO, United States; ^2^Biomedical, Energy and Materials, CFD Research Corporation, Huntsville, AL, United States; ^3^Physical Medicine and Rehabilitation Services, Department of Physical Therapy, James A. Haley Veterans’ Hospital, Tampa, FL, United States; ^4^School of Physical Therapy, Morsani College of Medicine, University of South Florida, Tampa, FL, United States

**Keywords:** vestibular hypofunction, older adults, rehabilitation games, motivation, engagement

## Abstract

**Introduction:**

Vestibular hypofunction is associated with dizziness, imbalance, and blurred vision with head movement. Vestibular rehabilitation is the gold standard recommendation to decrease symptoms and improve postural stability. The Clinical Practice Guidelines for vestibular hypofunction suggest home exercises 3–5 times daily, but patient adherence is a problem, with compliance rates often below 50%.

**Methods:**

An app was developed to increase engagement with home exercises by providing exercises as games. This study compared the accuracy of exercise performance in a one-time session using the app versus no-app and gathered participant feedback on using the app for vestibulo-ocular reflex (VOR) and balance exercises. The app was tested with 40 adults (20 women), mean age of 67 ± 5.7 years, with symptomatic unilateral or bilateral vestibular hypofunction. Participants completed VOR exercises in pitch and yaw planes, weight-shift, and single-leg balance exercises using an inertial motion unit to move the character on the tablet screen. Participants were randomly assigned to begin the exercises with or without the app.

**Results:**

Results show that during VOR exercises, participants achieved the prescribed frequency of head motion for the yaw plane (*p* ≤ 0.001) and reduced variability of head movement frequency in both the yaw (*p* ≤ 0.001) and pitch plane (*p* ≤ 0.001) in the app compared to the no-app condition. During weight-shifting exercises, a larger range of body motion was noted in the anteroposterior and mediolateral directions in the app compared to the no-app condition (*p* < 0.05). During single-leg balance exercises, pelvic motion was lower in the app versus no-app condition (*p* = 0.02). Participants modified their exercise performance and corrected their mistakes to a greater extent when they used the app during the VOR exercises. Participants agreed that they felt motivated while playing the games (97%) and felt motivated by the trophies (92%). They agreed that the app would help them perform the exercises at home (95%), improve their rehab performance (95%) and that it was fun to do the exercises using the app (93%).

**Discussion:**

The results of this study show that technology that is interactive and provides feedback can be used to increase accuracy and engagement with exercises.

## 1 Introduction

Data from the National Health and Nutrition Examination Survey (2001–2004) estimate that 35.4% of adults in the United States have vestibular dysfunction, and the incidence increases with age (1). More recent studies have confirmed these findings, showing that 34% of community-dwelling adults over 50 years of age who present with complaints of dizziness to primary care have a vestibular cause for their dizziness (2, 3). Uncompensated vestibular hypofunction is associated with symptoms of dizziness, visual blurring with head movement, and postural instability, which significantly increase the risk of falls (1, 4, 5). Vestibular rehabilitation is an exercise-based intervention to promote adaptation, habituation, and/or compensation for these functional deficits and has been proven to be effective in reducing dizziness, improving postural stability, improving confidence with mobility, and reducing fall risk (6–8). The Clinical Practice Guidelines (CPGs) for vestibular hypofunction provide evidence-based guidance for exercise prescription, including the optimal frequency, duration, and types of exercises to reduce symptoms and improve balance and mobility (9, 10). Based on the CPG, vestibular specialists typically provide home exercise programs in the form of handouts or treatment logs and recommend that patients perform these exercises between 3 and 5 times a day consistently and accurately (11). Unfortunately, less than 50% of patients with vestibular hypofunction report doing the exercises at home (12), and consistent completion of home exercises continues to be a challenge (11).

From the patient’s perspective, adherence to home-based vestibular exercises is difficult because the exercises are boring, they may not understand how to perform the exercises, they are afraid because symptoms of dizziness increase while doing the exercises, and a lack of feedback while exercising at home makes it difficult to determine if they are performing the exercises correctly (12–15). Due to these barriers, patients frequently stop doing their exercises, which can lead to poor outcomes. Technology-based exercise interventions have shown promising results in promoting adherence and engagement among older adults and in neurological populations such as stroke, multiple sclerosis, and Parkinson’s disease (16–21). However, technology-based solutions to increase engagement with vestibular exercises are limited. Hovareshti et al. (22) have developed the VestAid^™^ for service members and athletes after concussion injury, while Meldrum et al. (23) have created the VertiGenius^™^, which allows the prescription of individualized vestibular exercises but without the game-based feature.

To address the gaps in existing technologies available for treating vestibular dysfunction, we have developed a game-based app with vestibular exercises designed specifically for adults 65 years of age and older with dizziness. This app includes features such as a storyline, interactive characters, backgrounds with various degrees of visual stimulation, and game scores provided during the exercises. The app was designed to be easy for older adults to use on a mobile platform, such as a tablet. In a previous study, the prototype app was tested with healthy older adults, and the feedback obtained was used to make iterative changes in the app (24). Mistakes that were commonly made during exercises were identified, and machine learning algorithms were developed to detect mistakes in real-time and provide visual and auditory feedback during exercises to improve the accuracy of exercise performance.

The goal of this study was to test the revised app on adults over 65 years of age with unilateral or bilateral vestibular hypofunction in a one-time laboratory session. The primary aim was to compare the accuracy of exercise performance between the app and no-app conditions. The secondary aim was to assess patient perceptions and experiences with the app using validated questionnaires and open-ended questions.

## 2 Materials and methods

This was a single-arm prospective study that was approved by the Institutional Review Board at the University of Kansas Medical Center. The study was registered with clinicaltrials.gov (identifier NCT05436067).

### 2.1 Participants

Participants completed a screening form to make sure they met the study inclusion and exclusion criteria. In general, the participants who were recruited from the outpatient clinic completed the screening form in person, while those identified through database searches completed the screening form over the phone. The inclusion criteria were as follows: (1) between 60 and 75 years of age, (2) have dizziness or imbalance due to a diagnosed unilateral or bilateral vestibular hypofunction, which could be due to vestibular neuritis, labyrinthitis, or Meniere’s disease, (3) be able to give informed consent, (4) be able to read and speak English fluently, and (5) be able to get out of a chair independently.

The exclusion criteria were as follows: (1) pre-existing neurological diseases such as stroke, Parkinson’s disease, multiple sclerosis, or traumatic brain injury and (2) history of recent surgery or lower extremity musculoskeletal injury or pain that would impede performing the balance exercises.

Multiple sources were used for recruitment purposes, including (1) a hospital-based vestibular outpatient clinic, (2) the Healthcare Enterprise Repository for Ontological Narration (HERON) database, which is a search discovery tool to search de-identified data from the KU Health System medical records, (3) the Frontiers registry at the University of Kansas Health System, which includes people with a diagnosed vestibular disorder who have agreed to be contacted for research purposes, (4) the otolaryngology-head and neck surgery patient database of people with a diagnosed vestibular disorder, and (5) community referral.

### 2.2 Overall procedure

Human subject testing was performed in a laboratory setting at the University of Kansas Medical Center in a one-time session. All participants signed informed consent before the study began. Testing began with collecting demographic information, followed by a systems review. All participants completed the Dizziness Handicap Inventory (DHI), Activities Specific Balance Confidence Scale (ABC), and Visual Vertigo Analog Scale (VVAS) on the tablet using the VestRx™ (research version). The exercise program design was identical for all subjects, beginning with VOR X1 exercises in the yaw and pitch plane, followed by the weight-shift exercises (anteroposterior, mediolateral, and omnidirectional) and single-leg balance exercises. All participants completed the exercise program both with the “app” condition and without the app (the “no app” condition). The order in which each participant completed the conditions (app or no-app) was randomly assigned.

#### 2.2.1 Experimental setup

The app software was installed on an Android tablet, and the rehabilitation games were controlled by a MetaMotionR (MBientlab, Inc.) inertial measurement unit (IMU), connected to the tablet via Bluetooth. During the VOR games, the IMU was placed on the forehead using hypoallergenic adhesive tape, and IMU data on head motion obtained in the app were used to control the character in the rehabilitation games. During the weight-shifting and single-leg balance exercises, the IMU was secured to the waist using a gait belt so that pelvic motion controlled the character in the games. The tablet was mounted on a stand at arm’s length from the participant and at eye level for optimal use. Participants performed VOR exercises while seated in a comfortable chair, and weight-shift and single-leg balance exercises while standing behind the chair. During the app condition, the experimenter introduced the app to the participant; however, directions for exercises were mainly provided through the app. Before the start of each exercise, participants were asked to hold still so that the IMU was calibrated to the neutral position. Participants were encouraged to perform a practice game before the actual trial started. Each participant played one set of each game, and after each game, the participants could see their scores and trophies before moving on to the next game.

#### 2.2.2 Gaze stability VOR X 1 pitch and yaw (daring escape rehabilitation game)

**Figure 1:**
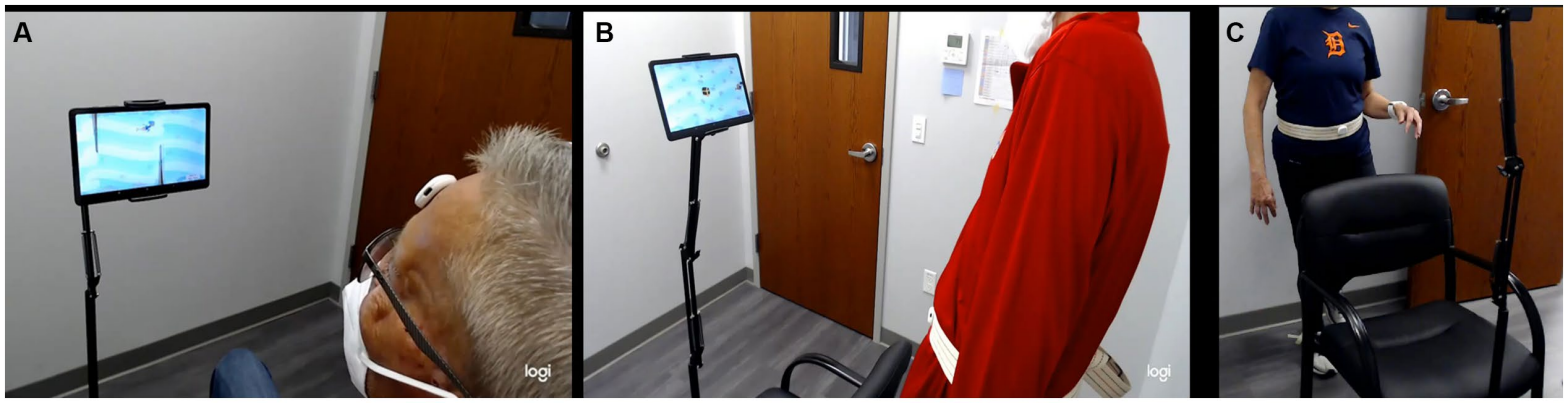
**(A)** Subject performing the VOR pitch exercise in sitting with the IMU on the forehead, **(B)** weight-shifting exercise in standing with the IMU at the waist, and **(C)** single-leg balance exercise with the IMU at the waist. VOR, vestibulo-ocular exercises; IMU, inertial motion unit.

Participants performed VOR exercises while sitting with an IMU attached to the forehead. The app software was preset to achieve 20 degrees of head range of motion from the midline, set at a frequency of 1 Hz, and a duration of 1 min. VOR exercises were started in the yaw plane, followed by the pitch plane, using the same parameters. Changes in the head range of motion were associated with a change in the movement of the character on the screen (measured by the IMU). Similarly, the angular velocity setting of head motion controlled the speed at which the obstacles appeared on the screen. This speed was calculated using the range of motion and the desired angular velocity to set the number of head rotations that needed to be made per second. In the “app” condition, participants controlled a character on the tablet screen by moving the head from left to right (yaw) or up and down (pitch) to avoid obstacles. Coins were awarded for each obstacle that was avoided. Participants received visual and auditory feedback from the app if they were moving their heads too far, too fast, or if they made jerky head movements. All participants completed the VOR games in the yaw and pitch directions at level 2 mode, which was a visually complex ocean-themed background (Figure 1A). In the “no app” condition, a letter was fixed to the wall, and the participant was instructed to move their head in the yaw and pitch plane for 1 min; however, no additional cues were provided.

#### 2.2.3 Weight shift (treasure hunter rehabilitation game)

Participants performed weight-shifting exercises in three directions: anteroposterior, mediolateral, and omnidirectional, while standing behind a chair. The participants performed weight shifting in each direction for 2 min with the IMU attached at the waist on a gait belt. In the “app” condition, the character on the tablet screen was controlled by the IMU, where leaning forward moved the character up, leaning backward moved the character down, and leaning left/right moved the character sideways. Coins were given as rewards if participants successfully shifted their body weight in the direction of the coin and returned it to the treasure chest. Instructions were given to move from the ankle and avoid bending from the hips or the knees (Figure 1B). In the “no app” condition, the participant was instructed to shift their body weight from side to side, front and back, and omnidirectionally for the specified time frame; however, no other cues were provided.

#### 2.2.4 Single-leg balance (balancing act rehabilitation game)

Participants performed single-leg balance exercises while standing with the IMU fixed at the waist. The participant performed this game twice: once standing on the left leg and once on the right leg for 45 s in each position. In the “app” condition, participants controlled the character on the tablet screen by remaining as stable as possible while balancing on a platform tilted laterally based on the movement of the IMU. Coins were awarded for maintaining a level pelvis. The participants were awarded either two, one, or zero coins depending on the degree of tilt at the pelvis (Figure 1C). In the “no app” condition, the participant was instructed to perform the single-leg balance exercise on each side; however, no cues were provided.

### 2.3 Post-exercise app evaluation questionnaires

After completing the “App” portion of the study, participants completed surveys to assess the ease of use of the app, enjoyment of the intervention, and friendliness of the tablet interface using the following questionnaires:

1. Perceived Usefulness and Ease of Use Questionnaire, which evaluates the ease of use of technology (25).
2. Usefulness, Motivation, and Enjoyment Questionnaire to evaluate the usefulness, motivation, and enjoyment of using the app (19).
3. User Interface Questionnaire, which evaluates the overall reactions to the software. This rates several reactions on a scale of 0 to 9 (with 5 being a neutral answer).

At the end of the entire session, participants were asked open-ended questions: (1) describe the positive aspects as you performed the exercises in the app, (2) describe the negative features as you performed the exercises in the app, (3) What changes would you like to see as you perform the exercises in the app? and (4) Are there any barriers that would prevent you from using the app at home?

### 2.4 Data processing

At the completion of each exercise session (with and without the app), raw IMU as well as summary data were encrypted and sent securely by the app to a cloud storage database in a de-identified format. Data were retrieved from the database and analyzed using custom-written code in Python.

#### 2.4.1 Gaze stability VOR X 1 pitch and yaw

The roll, pitch, and yaw angle of the head were calculated using the calibration trial at the start of each game as the neutral head position (the participant was asked to hold their head steady during calibration while looking at the center of the tablet screen). This first step of the analysis was performed within the app. A peak-finding algorithm was used to identify the peak left and right head angles of each head rotation. The head range of motion (ROM) was calculated as the average distance between consecutive peaks, and the frequency of the peaks was calculated for the yaw and pitch direction.

#### 2.4.2 Weight shift

The mediolateral (ML) and anteroposterior (AP) angles of the pelvis were calculated using the calibration trials at the start of each game as the neutral stance position. This first step of the analysis was performed within the app. Peak excursions of the pelvis were identified using a peak-finding algorithm. The pelvis range of motion (ROM) was calculated as the average distance between consecutive peaks, and the frequency of the peaks was calculated. This was calculated for the ML and AP directions for the respective games.

#### 2.4.3 Single-leg balance

The mediolateral (ML) and anteroposterior (AP) angles of the pelvis were calculated using the calibration trials as the neutral stance position at the start of each game. This first step of the analysis was performed within the app. A resultant angle of the ML and AP angles was calculated to represent the overall excursion of the pelvis. Peak excursions of the pelvis were identified using a peak-finding algorithm on the resultant angle data. The fluency of the pelvic motion was calculated to represent the smoothness of the pelvic motion during the single-leg balance games. Fluency was calculated using methods previously described (26). A larger fluency value indicates a smoother motion.

## 3 Statistical analysis

Statistical analysis was performed using code written in Python. Data were tested for normality using a normality test in the SciPy module that is based on D’Agostino and Pearson’s (27, 28) test that combines skew and kurtosis to produce an omnibus test of normality. The statistical difference between the app and no-app conditions was tested using a paired t-test with an alpha value of 0.05 for data that were normally distributed (VOR pitch and yaw range of motion and frequency), and a Shapiro–Wilcoxon test (non-parametric) for paired observations was used for data that were not normally distributed (weight-shift exercise range of motion and frequency for mediolateral and anterior-posterior exercises, single-leg balance range of motion and fluency).

## 4 Results

**Figure 2:**
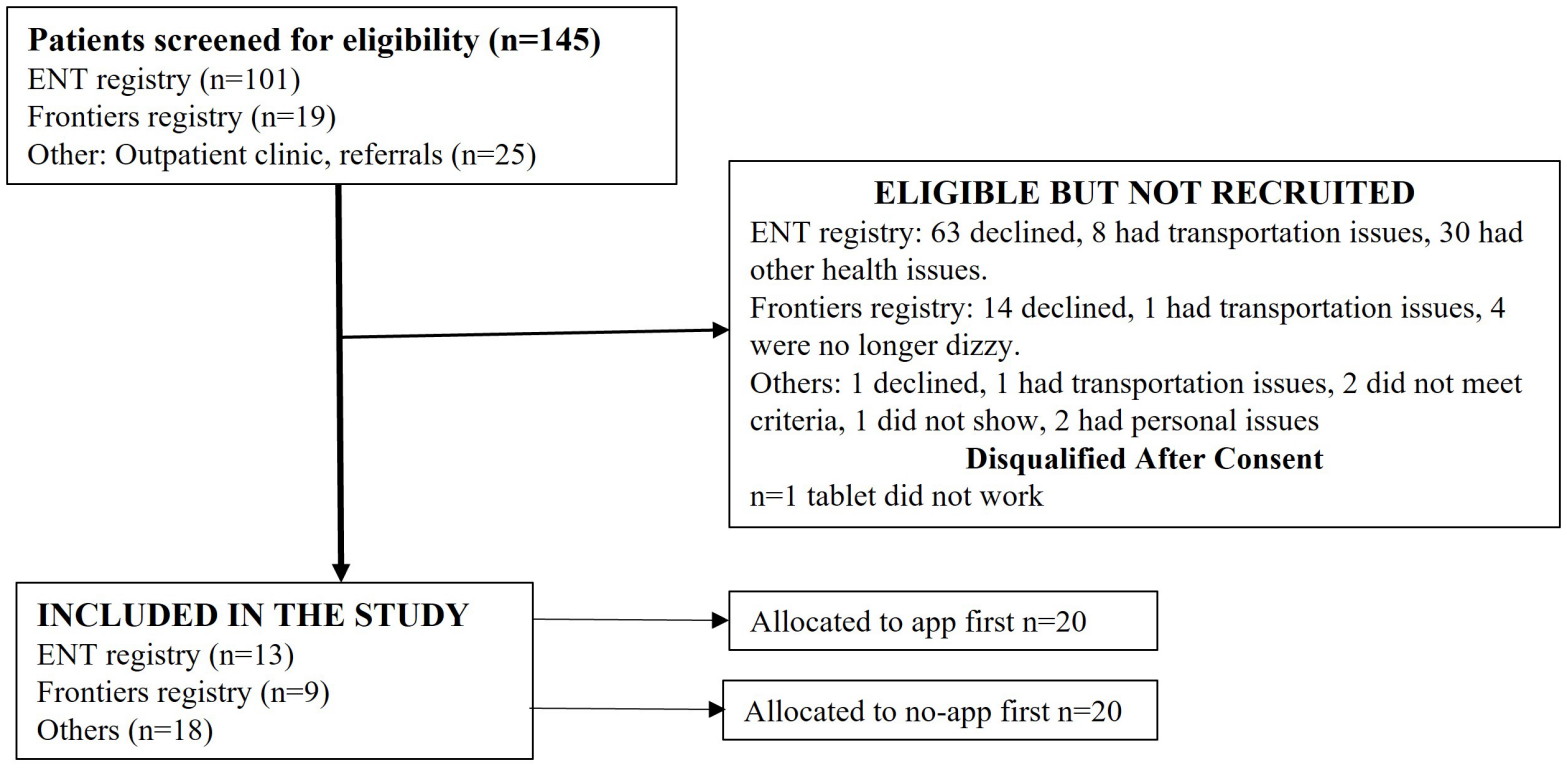
Participant recruitment.

The app was tested with 40 adults (20 women), mean age of 67 ± 5.7 years, with unilateral or bilateral vestibular hypofunction. The participant flow diagram depicts the subjects screened, excluded, and enrolled (Figure 2). No adverse events, such as falls, were noted during testing. The mean total dizziness handicap scores were 31.9 ± 24.5 (range 2–100), and the mean total activities specific balance confidence scores were 78.19 ± 23.4 (range 20–98.8). Similarly, a wide range was seen in the VVAS scores from 0 to 10, but for most participants, the range of VVAS scores was between 0 and 3.

### 4.1 Accuracy of exercise performance with and without the app

#### 4.1.1 VOR exercises

**Figure 3:**
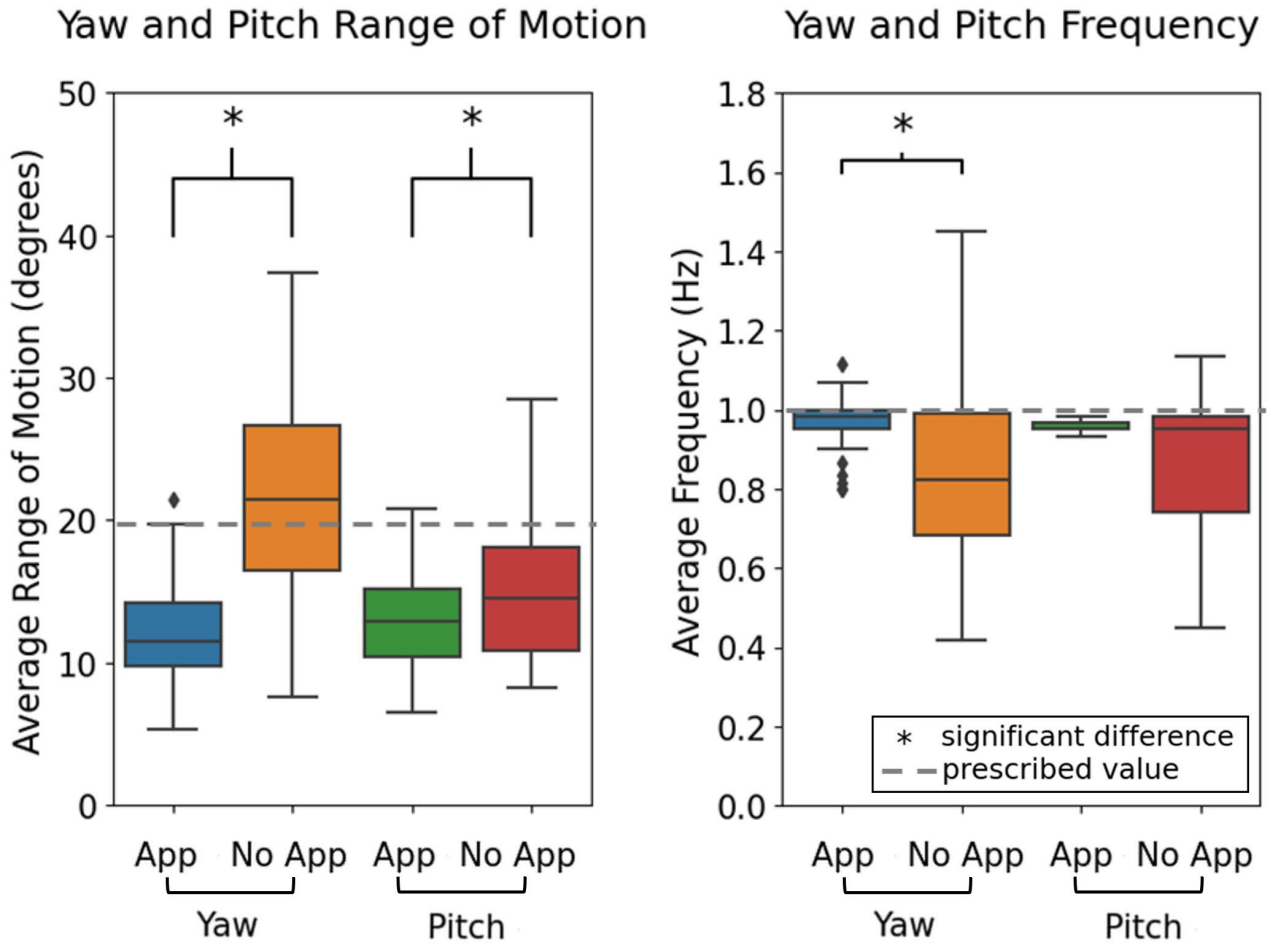
Comparison of range of head motion and frequency of head motion in the app and no-app conditions. Dotted line represents the parameters set within the games, where the head range of motion was set to 20 degrees from the midline and the frequency of head motion was set at 1 Hz. ^*^Indicates significant differences between groups, with alpha set at 0.05.

The range of head motion was significantly different between the app and no-app conditions for the yaw and pitch VOR exercises (*p* ≤ 0.001 and *p* = 0.02, respectively), with a smaller range of motion in the app condition. Variance in range of motion was significantly smaller when using the app for the yaw VOR exercise (*p* = 0.04), while for the pitch VOR exercise, variance in range of motion was not significantly different between the app and no-app conditions (*p* = 0.12). The frequency of head motion was significantly different between the app and no-app conditions for the yaw but not for the pitch VOR exercises (*p* ≤ 0.001 and *p* = 0.06, respectively), with a higher frequency when using the app. The variance in the frequency of head motion was significantly smaller when using the app for both the yaw and pitch VOR exercises (*p* ≤ 0.001 and *p* ≤ 0.001, respectively) (Figure 3).

#### 4.1.2 Weight-shifting exercises

**Figure 4:**
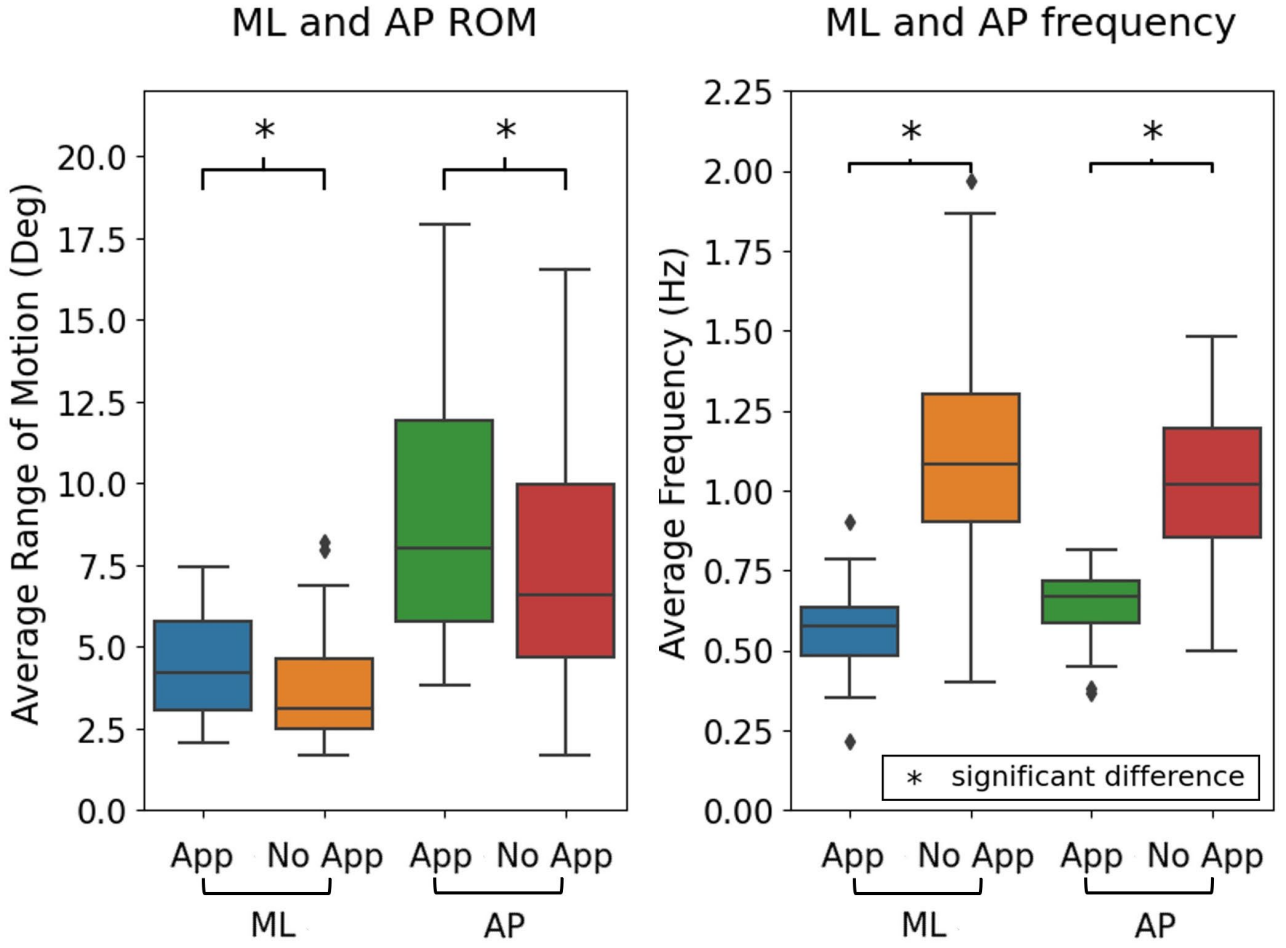
Comparison of range of body motion and frequency of motion during the weight-shifting exercises in the app and no-app conditions in the ML and AP directions. ML, mediolateral; AP, anteroposterior; ROM, range of motion.

The range of motion of the body was significantly different between the app and no-app conditions for the ML and AP weight-shift exercises (*p* = 0.04 and *p* = 0.01, respectively), with a larger range of motion when using the app. Variance in range of motion was not significantly different between the app and no-app conditions for both the ML and AP weight-shift exercises (*p* = 0.93 and *p* = 0.81, respectively). The frequency of whole-body motion was significantly different between the app and no-app conditions for the ML and AP exercises (*p* ≤ 0.001 and *p* ≤ 0.001, respectively; Figure 4), with a lower frequency when using the app. The frequency of body motion was significantly less variable when using the app for both the ML and AP weight-shift exercises (*p* ≤ 0.001 and *p* ≤ 0.001, respectively) (Figure 4).

#### 4.1.3 Single-leg stance exercises

**Figure 5:**
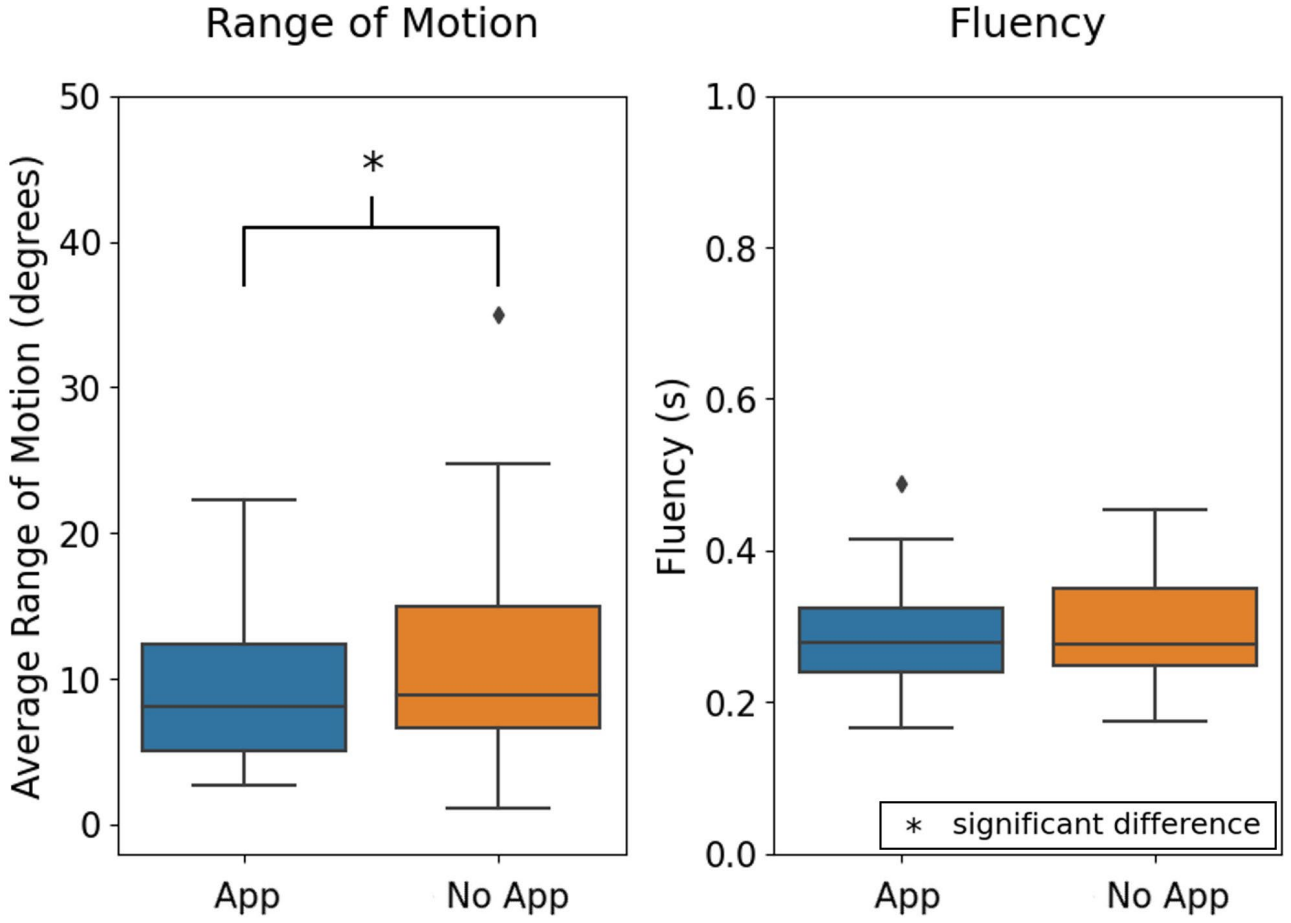
Comparison of range of motion and fluency of motion as an aggregate of right and left single-leg stance. ^*^Indicates significant differences between groups, with alpha set at 0.05.

The resultant range of motion of the body was significantly different between the app and no-app conditions with single-leg stance, with less motion observed in the app condition (*p* = 0.02); however, the fluency of motion was similar in both conditions (*p* = 0.26) (Figure 5).

**Figure 6:**
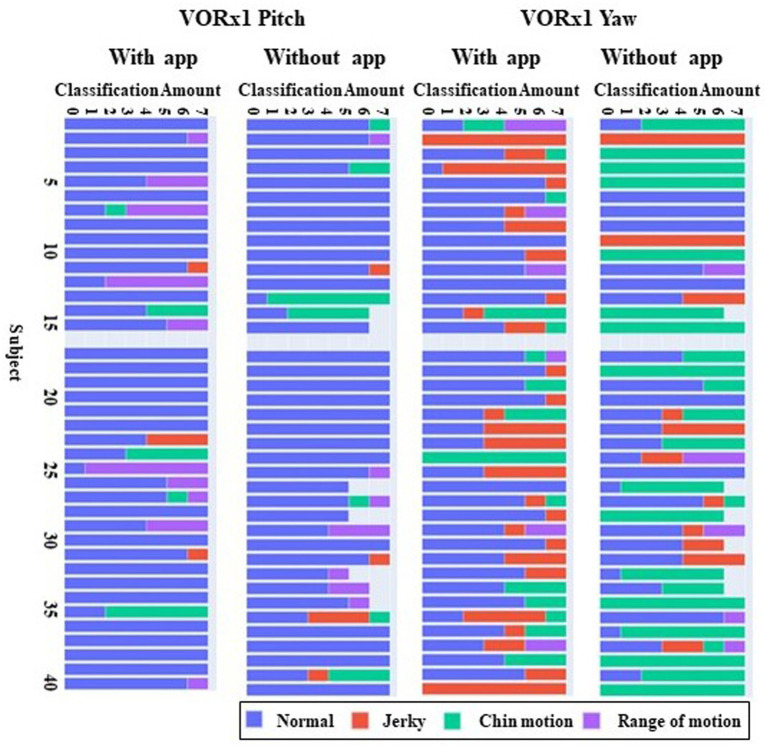
Common errors made during VOR exercises with and without the app in the pitch and yaw planes. Errors were identified every 7 s over the 60 s duration of VOR exercises in the pitch and yaw plane. The horizontal bars represent each individual participant, and the color codes indicate the mistakes that were made by that participant in each 7 s time window. VOR, vestibulo-ocular reflex.

When using the app to perform the VOR exercises, the most common errors identified by machine learning algorithms were jerky head motion observed in 13% of participants, excessive chin motion identified in 7%, and range of head motion past 20 degrees from midline observed in 9% of participants. In the no-app condition, the errors detected were excessive head motion in 28%, jerky head motion in 4%, and excessive chin motion in 2% of participants (Figure 6). Based on the errors identified, auditory and written feedback on the screen were provided to subjects when they were using the app for VOR exercises (Table 1).

**Table 1:** Errors made and corrected during exercises in the app and no-app conditions.

		App	No-app
VOR pitch exercise	Errors made	42%	28%
	Errors corrected	21%	10%
	Error not corrected	21%	18%
VOR yaw exercise	Errors made	93%	80%
	Errors corrected	44%	18%
	Error not corrected	49%	62%

The percentage of trials during the VOR pitch and yaw exercises with and without the app shows the number of errors made in each condition, the number of errors corrected, and the number of errors not corrected.

### 4.2 Participant perceptions and experiences with the app

#### 4.2.1 Results of the perceived usefulness and ease of use questionnaire

**Figure 7:**
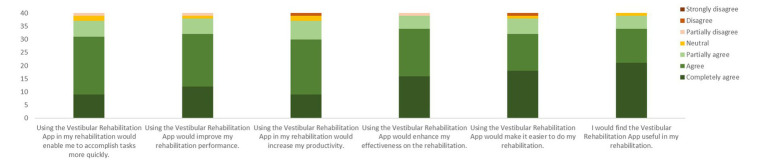
Responses to the six questions on the perceived usefulness of the VestRx. The color bars are cumulative responses per category (ranging from completely agree to strongly disagree), summing up to the total number of 40 participants.

**Figure 8:**
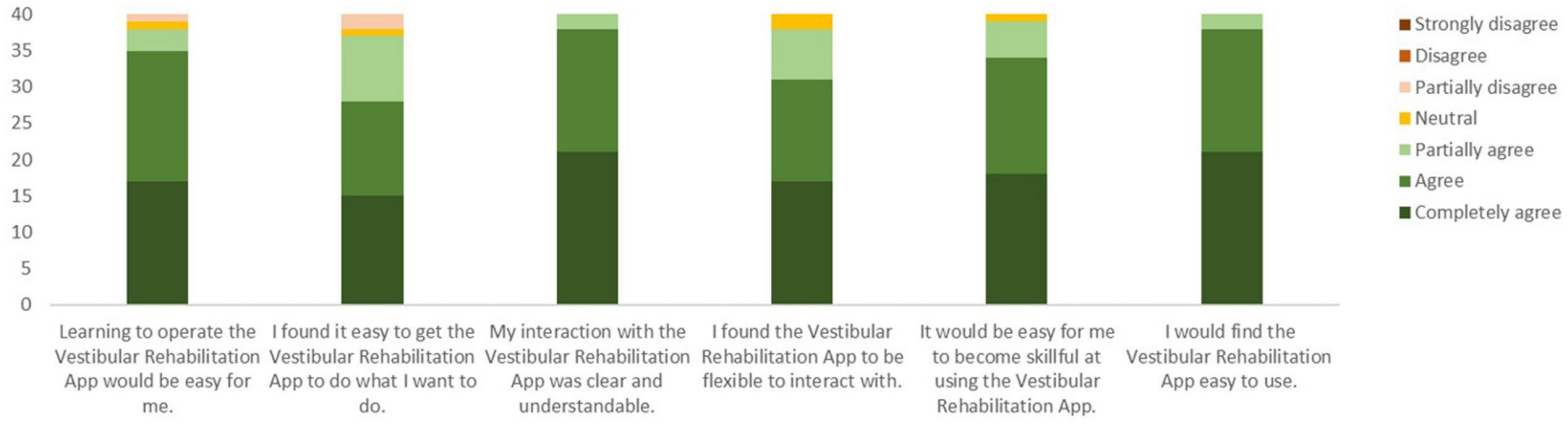
Responses to the six questions on the perceived ease of use of the VestRx. The color bars are cumulative responses per category (ranging from completely agree to strongly disagree), summing up to the total number of 40 participants.

More than 90% of the participants agreed that the app would help the rehabilitation process and allow them to accomplish their goals more quickly (Figure 7). More than 90% of participants agreed or partially agreed that interacting with the app was easy (Figure 8).

#### 4.2.2 Results of the usefulness, motivation, and enjoyment questionnaire

**Figure 9:**
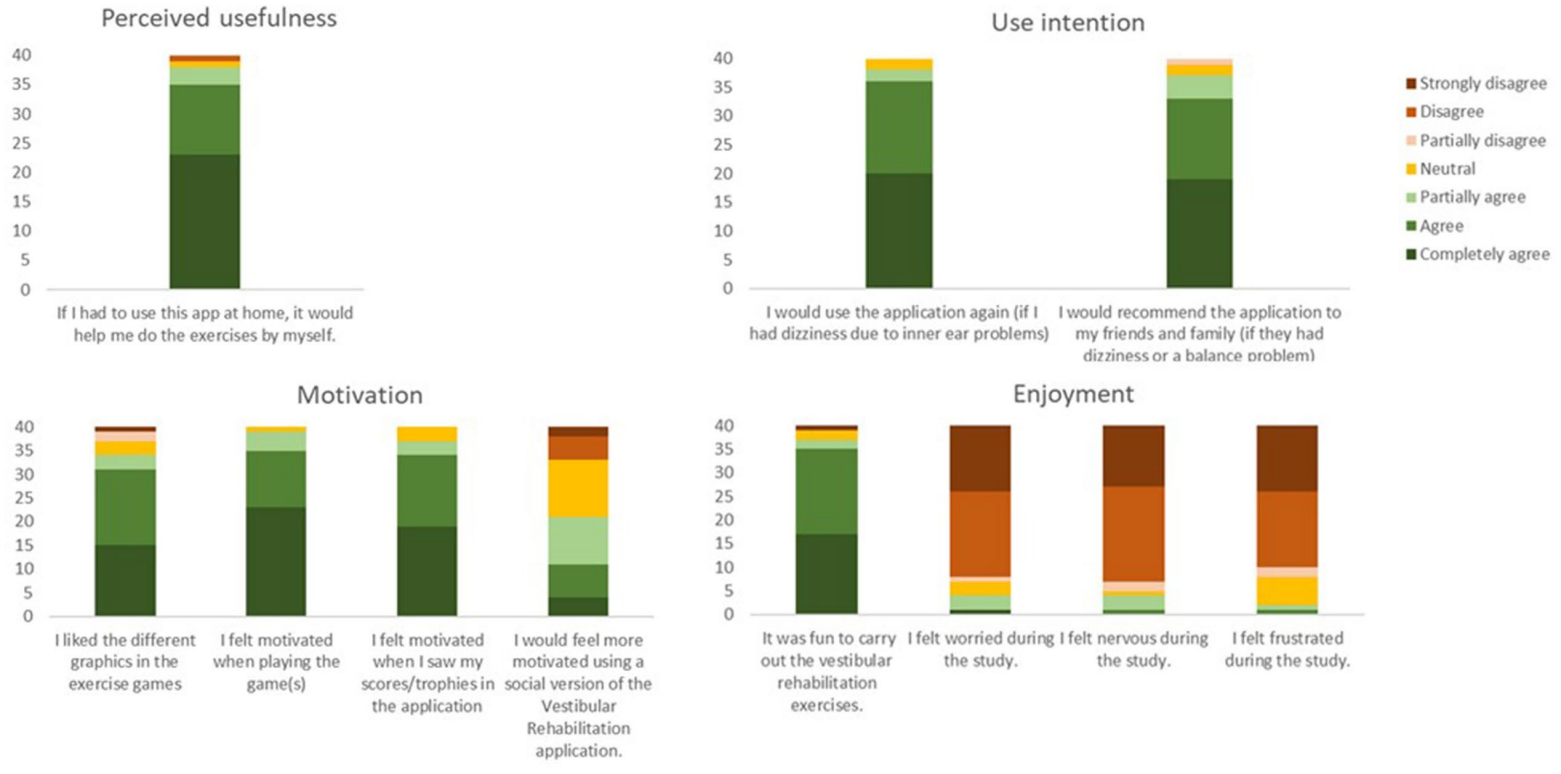
Overview of responses obtained from the usefulness, motivation, and enjoyment questionnaire. The color bars are cumulative responses per category (ranging from completely agree to strongly disagree), summing up to the total number of 40 participants.

More than 90% of participants agreed that they would use the app or recommend it to others with dizziness. Although 97% felt motivated by the trophies and scores provided, only 50% felt they would be motivated by a social version of the app. Ninety-two percent of participants agreed that using the app was fun; however, 10% felt worried, nervous, and frustrated when using the app (Figure 9).

#### 4.2.3 User Interface questionnaire

**Figure 10:**
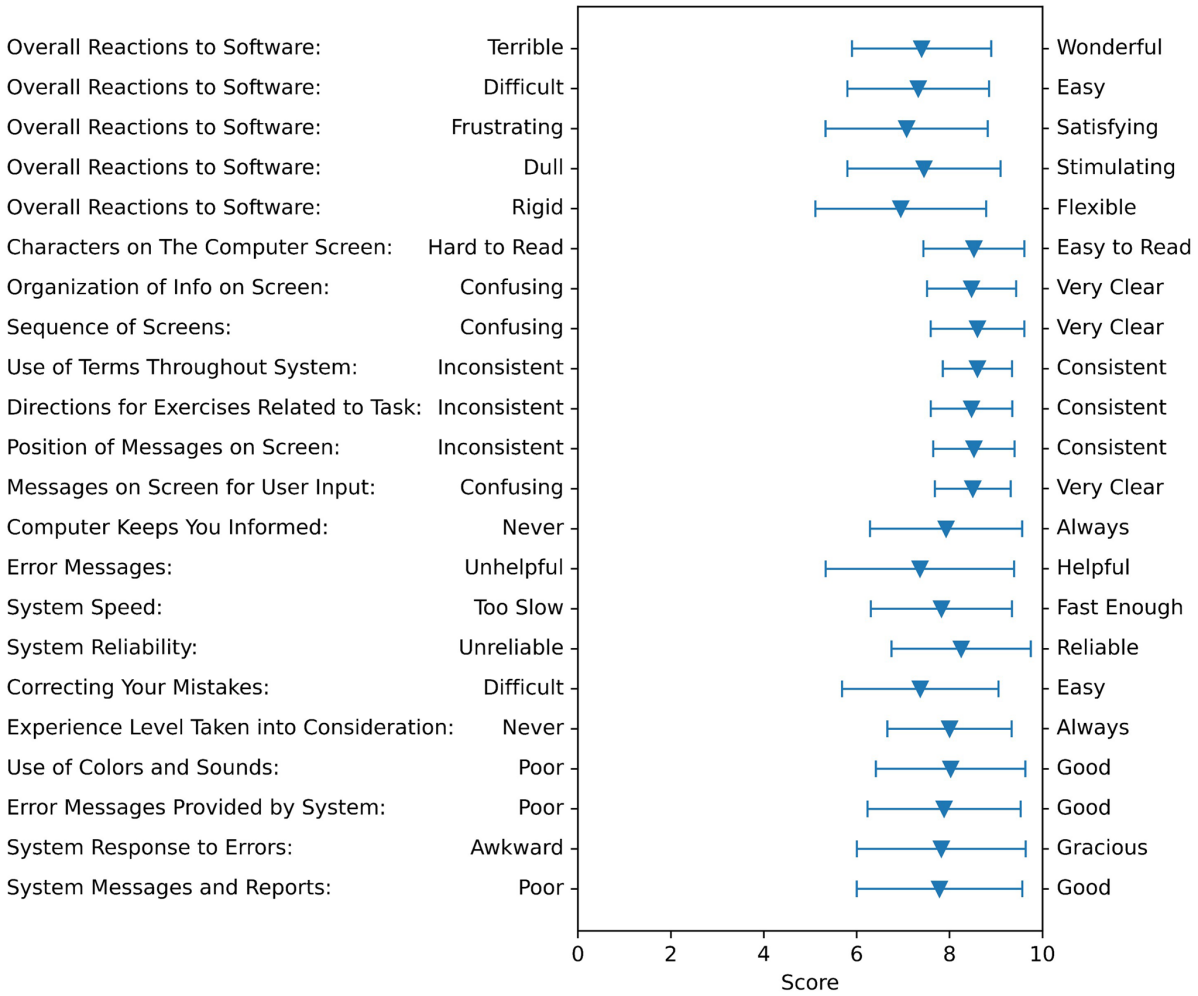
Feedback summary on the user interface questionnaire. The triangles show the averages of all subjects, and the bar shows the standard deviation. This questionnaire is rated on a scale from 0 to 9, with 0 being the worst possible, 5 being neutral, and 9 being the best possible.

Average ratings for each item in the User Interface Questionnaire are presented in Figure 10. Average responses were all at 7 or higher on a scale of 0 to 9.

#### 4.2.4 Responses to open-ended questions

Open-ended questions were recorded, transcribed, and categorized into various themes. Complete responses are provided as Supplementary material.

##### 4.2.4.1 Describe the positive aspects as you performed the exercises in the app

Interactive and engaging: “Well, it was interacting to the point that you could actually see what you were accomplishing or not accomplishing the effect and how diminished your senses and ability to be mobile were” (subject #12).

Feedback provided: “I like the feedback. I like the fact that monitors where you are moving, how you are responding to the exercises. I also thought was kind of unique in that the further I moved, the faster it went. So, the response of the system from that was great” (subject # 28).

##### 4.2.4.2 What are the negative features as you performed the exercises in the application?

Sensor response time: “I did not feel that they responded quickly enough to some of the moves that I made. I actually had emphasis where I was leaning this direction you know the figure was moving out that direction before it finally turned around and came back so that was the one thing” (subject #12).

##### 4.2.4.3 What changes would you like to see to help you perform the exercises in the application?

Auditory and written feedback: “I prefer it talking to me, rather than the writing, because if I was doing the exercise and I knew I was doing it for so many minutes and I was supposed to be doing it I would have to stop the exercise to read the feedback whereas if I was doing it, and it talked to me. It did not interrupt my flow” (subject #4).

##### 4.2.4.4 Are there any barriers that would prevent you from using the vestibular rehabilitation app at home?

Equipment: “I would say that I would be concerned for some people. I mean, most people have a phone, but not everybody has a tablet” (subject #36) and “Yeah, I guess the only barrier is to always have a stand” (subject #6).

## 5 Discussion

Vestibular rehabilitation is the gold standard treatment for people with a diagnosed unilateral or bilateral vestibular hypofunction. It is an exercise-based rehabilitation program that includes adaptation, habituation, and compensation components. Unfortunately, in general, adherence to and engagement with vestibular exercises is poor, leading to less-than-optimal improvement in balance, gait, and symptoms of dizziness. The aims of this study were to determine if performing exercises using an interactive gaming app improved the accuracy of exercise performance compared to conventional therapy and if the gaming and interactive features of the app had the potential to increase engagement with the exercise program. We found that when the app was used to do the VOR (daring escape) exercises, the frequency of head motion was close to the prescribed frequency compared to without the app. Similarly, the balance games, treasure hunter, and balancing act were performed more consistently, as measured by reduced variance in the frequency and range of body motion compared to the no-app condition. Participants found the games to be interesting, the feedback during the games to be useful, and the scores and trophies to be motivating. The results of this study provide a characterization of exercise performance with the app in comparison to conventional therapy and provide information on whether patients are likely to enjoy the app and use it as prescribed by a clinician.

Exercise apps have become an integral part of rehabilitation, and technology can provide various benefits when incorporated into exercise programs. One area where technology can be harnessed to assist during vestibular exercises is to provide feedback during exercises. People with vestibular hypofunction often stop doing their exercises because they are unsure if they are doing the exercises correctly. The app incorporates machine learning algorithms to provide augmented feedback during exercises. Feedback is a vital aspect of motor learning, and learning is enhanced when practice includes a variety of tasks while receiving feedback (29, 30). The app provides two types of feedback: first, knowledge of performance, which is provided through explicit auditory and written feedback on the tablet screen when a mistake is made; and second, knowledge of results, which is terminal feedback about the goal achieved. Multimodal feedback is known to increase sensory enhancement, which allows for increased engagement (31); however, we need to consider the potential overload of the auditory and visual feedback channels. Based on the feedback obtained from participants, reading the visual feedback during the VOR exercises was difficult; therefore, further studies that specifically target the best feedback mode in this patient population are required.

Other apps currently available on the market have used feedback to increase engagement during vestibular rehabilitation. VestAid uses a metronome to provide feedback on the speed of head movements and offers a gamified feedback mode with rewards to keep patients engaged, motivated, and compliant with therapy; however, it does not provide real-time feedback during the exercises on accuracy of performance (22). VertiGenius provides auditory feedback using a metronome during gaze stabilization exercises and provides real-time visual feedback during gaze stabilization exercises through a traffic light system. Participants found the visual feedback, in the form of the traffic light system, helpful because it corrected the frequency of head movement during exercises (23). The VestRx does not use a metronome; instead, the frequency of head movement is entered by the clinician in the app portal, and the app presents the obstacles to achieving the prescribed frequency.

We observed that the feedback provided allowed participants to correct the errors made. In a previous study, we identified errors that were commonly made during VOR exercises (24). Machine learning algorithms were trained to identify these errors and provide auditory and written feedback explicitly stating the type of error being made. Twenty-one percent of participants corrected their errors when they used the feedback provided by VestRx for VOR pitch exercises, compared to only 10% who corrected errors in the no-app condition. In the yaw plane, 44% of participants corrected mistakes during app use compared to 18% who corrected their mistakes in the no-app condition. The opportunity to correct mistakes can improve the accuracy of exercises, increase engagement, and motivate patients to improve their scores. Overall, using the app increased the accuracy of the daring escape VOR exercises where the frequency of head motion was close to the prescribed frequency of 1 Hz and the variability of head motion was decreased; however, the range of head motion was significantly smaller in the yaw plane compared to the prescribed 20 degrees in each direction from midline. Based on this observation, the algorithms have been re-calibrated to ensure that head movement range is increased in the yaw and pitch planes during the daring escape game. Similarly, the range and frequency of pelvic motion during the treasure hunter weight-shift game showed less variability and a lower frequency of pelvic motion, indicating that participants were moving in a deliberate manner and holding the end-of-range positions for a longer period of time. These positions are most challenging for patients; therefore, they are usually rushed through. The app provides the necessary feedback to hold those difficult positions for a longer time.

Technology also has great potential to increase engagement and motivation to perform exercises, as we have seen in the responses to the open-ended questions and the questionnaires used in the study. Valenzuela et al. (21) have shown that technology-based interventions have higher adherence rates due to the reported enjoyment of using these programs, indicating that enjoyment is crucial to developing a successful program. Understanding the barriers and motivators in each patient population provides a better understanding of their specific needs to create apps that are personalized for them, as indicated by Schootemeijer et al. (32). The needs of people with vestibular hypofunction are unique. Driving to and from the clinic can increase symptoms of dizziness; therefore, having the option to use the app for home-based exercises is important to them. VOR exercises, which are prescribed to decrease symptoms of dizziness and gaze instability, can inherently increase symptoms further; therefore, driving home after performing a vestibular session in the clinic may be difficult. Having the option to do the exercises at home with regular monitoring by the vestibular therapist can reduce some of these barriers. The VestRx can be used to prescribe individualized exercises, and the response to the exercises can be monitored remotely to modify the prescription. The VVAS is built into the app as a sliding scale from 0 to 10 to be completed before and after each VOR exercise. Although the VVAS was not used for this study, our next step is to trial the app in the patient’s home and use the VVAS for remote monitoring to change the exercise prescription based on the patient’s response to the exercises.

Overall, we found that participants enjoyed using the app; they were independent with using the app in the lab setting and reported that the app would help them do the exercises at home. It was interesting to note that a few participants reported that doing the exercises in the app made them anxious; therefore, education about the different features and games in the app is essential to reducing their fears. Participants provided detailed feedback on the games, the graphics, barriers, and facilitators to using the app. The feedback received will be incorporated into the next iteration of the app, and the barriers described to using the app at home will be considered in the home-based exercise trial, which will be conducted next.

### 5.1 Limitations

One limitation of the study is that the exercises were not individualized for each patient. Our purpose was to determine if using the app changes the accuracy and performance of exercises; therefore, the study was designed to provide identical exercises with the same parameters of frequency and duration. The participant group was heterogenous, with a large range in the DHI, ABC, and VVAS scores. Therefore, the intensity of exercises may have been too easy for some and too difficult for others. Participants were randomized to start with either the app or no-app conditions; however, those participants who used the app first may have performed better in the no-app condition. Future studies examining the effect of corrective lenses while performing exercises are needed since patients may change how they move their heads when wearing glasses, which may be detected as an error by the ML algorithm. The ML algorithms may need to be re-trained for people who wear glasses and are part of our ongoing efforts to increase the sensitivity of the app to address the needs of a larger population. Another limitation of the version of the app used for this study is the use of the IMU, which requires people to store, charge, and correctly place it on their bodies. Our continuing study includes exploring video-based marker-less motion capture to reduce barriers to compliance due to hardware requirements.

## 6 Conclusion

The use of technology to provide vestibular rehabilitation exercises for individuals with uncompensated unilateral or bilateral hypofunction holds great potential. The game-based app, VestRx, has an interactive storyline, engaging characters, and motivating elements such as scores and trophies. The use of the app improved the performance of vestibular rehabilitation exercises, and explicit feedback provided during the session enabled participants to correct the errors they made. Although these features can increase motivation and engagement with exercises, other factors such as finding the time to do the exercises, not seeing immediate results, and remembering to do the exercises need to be considered. Providing education on the importance of exercises, limiting the duration of the exercises, and recommending strategies to alleviate the increase in symptoms can further increase adherence and reduce fears associated with vestibular exercises.

## Data availability statement

The raw data supporting the conclusions of this article will be made available by the authors, without undue reservation.

## Ethics statement

The studies involving humans were approved by University of Kansas Institutional Review Board. The studies were conducted in accordance with the local legislation and institutional requirements. The participants provided their written informed consent to participate in this study. Written informed consent was obtained from the individual(s) for the publication of any potentially identifiable images or data included in this article.

## Author contributions

LD’S: Conceptualization, Data curation, Formal analysis, Investigation, Methodology, Project administration, Resources, Supervision, Writing – original draft, Writing – review & editing. TP: Data curation, Formal analysis, Investigation, Methodology, Writing – review & editing. KP: Data curation, Formal analysis, Investigation, Methodology, Writing – review & editing. NP: Conceptualization, Data curation, Formal analysis, Resources, Visualization, Writing – review & editing. KM: Methodology, Software, Validation, Writing – review & editing. TZ: Methodology, Software, Validation, Writing – review & editing. MR: Software, Validation, Writing – review & editing, Methodology. KS: Methodology, Writing – review & editing. PR: Conceptualization, Data curation, Formal analysis, Funding acquisition, Investigation, Methodology, Project administration, Resources, Software, Validation, Writing – review & editing.
