# Supplementary material for: A gaming app developed for vestibular rehabilitation improves the accuracy of performance and engagement with exercises
Source: Front Med (Lausanne). 2023 Nov 24;10:1269874. doi: 10.3389/fmed.2023.1269874 (PMC10704144; doi:10.3389/fmed.2023.1269874)
Supplement: Supplementary file 1 [file Table_1.DOCX]

Supplementary Material

**VestRx improves accuracy of performance and engagement with exercises during vestibular rehabilitation**

# Supplementary Table

Detailed responses from participants obtained based on the open-ended questions. Participant responses were recorded, transcribed, and categorized based on themes that emerged.

**Question #1: Describe the positive aspects as you performed the exercises in the app,**

| Motivation |  |
| --- | --- |
| #1, age 65 (line 7) | “Motivates you to get better” |
| #1, age 65 (line 7-9) | I think it motivates you to try to get better. You know, it's easier to see “oh gosh, I could improve my score”. So, it makes you want to do your therapy more frequently I think also. |
| #16, age 60 (line 659) | “I could see that I can see it helping motivate.” |
| #22, age 61 (line 825-826) | “I also liked that it did scoring because apparently I'm motivated by that - I want to know that I got everything” |
| #27 age 64 (line 943-944) | “I think that I would be more inclined to do the exercise because it's not as much just standing there like that one was – you're interacting.” |
| #31 age 65 (line 1019) | “It would motivate me to do it, to try and beat my score - It would motivate me to do it.” |

| Engagement/fun |  |
| --- | --- |
| #2, age 71 (line 27) | “Gives me something to focus on” |
| #3 age 62 (line 60) | “As long as you’re having fun doing something, it’s easier” |
| #4 age 57 (line 122) | “It was fun” |
| #8 age 75 (line 270-271) | “Learning to do the different movements to control the thing on the screen. I guess that was enjoyable too” |
| #8 age 75 (line 277-279) | “It was more, you were accomplishing something with the app than when you were just doing the things without, but the app made it that you were accomplishing things. Made it more interesting I guess.” |
| #9 age 73 (line 334-335) | “Well, it was more fun. So you'd want to do it longer for one thing, you know because I get bored with the looking at the letter thing. So it was more fun.” |
| #11 age 74 (line 418-419) | “It was very video game liked and for people who are into video games, that would be absolutely natural for them, I’m sure.” |
| #12 age 70 (line 457-459) | “Well, there was- It was interacting to the point that you could actually see what you were accomplishing or not accomplishing the effect and how diminished your senses and ability to be mobile were” |
| #15 age 69 (line 614-616) | “The positive features were actually it was kind of fun. I liked the, you know, the cartoon type character to it. It was fun for me. I liked all the colors of it so visually stimulating, I would say that was the biggest thing for me.” |
| #16 age 60 (line 658) | “That you get these things that you’re asked to do to help yourself and they are made more fun” |
| #20 age 71 (line 806) | “I thought it was challenging- fun and challenging.” |
| #22 age 61 (line 826-828) | “It's not boring, so I think that's helpful. Even when we were doing the side to side of the standing, or whatever, it was better to be doing it with the app than to be doing that without it.” |
| #25 age 61 (line 889) | “The games, they're very positive. It’s actually competitive. I thought it was very helpful.” |
| #39 age 71 (line 1176-1177) | “It wasn't as boring. That comes to mind as the big thing. As far as looking around in the background and stuff and you know it's just different - It's not as boring as just looking at the letter and doing it.” |
| #40 age 69 (line 1197-1198) | “I think the game aspect of it and the animations are all interesting. It's pretty simple animation, but it does just jazz things up a little bit.” |

| Ease of use |  |
| --- | --- |
| #6 age 63 (line 202-204) | “Well, it was easy to use. I thought it was pretty smooth. I was kind of worried it might be kind of jerky or something like that. And it was pretty easy to learn to make it do what you were supposed to be doing.” |
| #13 age 73 (line 499-500) | “Easy to use. It seemed very easy to use and I mean, it was pretty clear what you know what they wanted you to do.” |
| #17 age 72 (line 695) | “I thought it was easy to use and pretty clear what they wanted you to do” |
| #18 age 74 (line 712) | “I think it was the easy part of using it, the idea that you know you can use it” |
| #22 age 61 (line 830-831) | “It makes a big difference because 45 seconds with versus 45 without - It was easier with for sure.” |
| #24 age 60 (line 868-869) | “The explanations on how to do everything was very straightforward. Tells you exactly how to do it. It's pretty simple so.” |
| #29 age 64 (line 983-985) | “They weren't too hard, which I thought was kind of good because I know that sometimes they can be -well, when I've seen my kids play them - So I thought they were easy to do, but challenging, but not too challenging.” |
| #32 age (line 1053-1054) | “I like the setup of it. I think it's pretty, it's simple, it's straightforward, there's no real guessing to it, the directions are clear.” |
| #36 age 69 (line 1134-1135) | “To the point - there's not a lot of extraneous information to slow you down or to make you get bored and just start scrolling through screens.” |
| #38 age 64 (line 1162) | “It was easy to use. I guess it wasn’t laborious, you know it. Was fairly easy to use.” |

| Feedback |  |
| --- | --- |
| #2 Age 71 (line 27, 28) | “Gives me feedback while I’m doing it, just to see if I’m doing it appropriately” |
| #5 age 67 (line 164-165) | “I liked the good feedback and the error messages, and the health plans. I mean just the overall feedback you got from it.” |
| #14 age 67 (line 547-549) | “Well, I liked it because they told you that you went too high or you went too low those kind of things and. And they had a number there and they had a score that. That was kind of cute too, I felt like it was like something I wouldn’t have thought about but they did.” |
| #28 age 70 (line 958-960) | “I like the feedback. I like the fact that monitors where you're moving, how you're responding to the exercises. I also thought was kind of unique in that the further I moved, the faster it went. So, the response of the system from that was great.” |
| #35 age 72 (line 1095) | “That it lets you know what you need to work on. The feedback that they gave you.” |
|  |  |

**Question #2: Describe the negative features as you performed the exercises in the app**

| Description/instruction of exercises |  |
| --- | --- |
| #2 age, (line 30-31) | At times it didn't explain well what it was going to have me do but the practice gave me the option of trying it out. |
| #6 age 63, (line 206) | “The redundant positioning of the sensor and stuff like that.” |
| #11 age 74 (line 421-422) | “Well, when you move from collecting the coins to just the balance thing, it was a totally different orientation, it took a little getting used to” |
| #32 age 68 (line 1056-1057) | “Sometimes in the directions part, though in the verbiage of it, there were a couple slides that they could have done away with because it was a little too wordy.” |

| Sensor/calibration issues |  |
| --- | --- |
| #8 age 75 (line 281-284) | “the big sensor problem, not moving in the right directions and everything. I had it going one way and then I would switch to get it to come back and it would be going the other way. That was the only thing that was, it was something I would be wanting to play with to see if it was actually me or the app that was causing the problem.” |
| #12 age 70 (line 464-466) | “I didn't feel that they responded quickly enough to some of the moves that I made. I actually had emphasis where I was leaning this direction you know the figure was moving out that direction before it finally turned around and came back so that was the that was the one thing.” |
| #22 age 61 (line 833-834) | “I guess the one time the mermaid wasn't moving fast enough for me.” |
| #26 age 74 (line 928) | “The slowness.” |
| #31 age 65 (line 1021) | “Sometimes a little bit too fast than that.” |
| #40 age 69 (line 1200-1201) | “in some cases with the little person - going up and down was harder and left or right, and the functionality of the ability to move things around. Sometimes it was frustrating.” |

| Font size/graphics |  |
| --- | --- |
| #16 age 60 (line 662-663) | “That goes back to being little. I guess it depends on how big your screen is” |
| #24 age 60 (line 873-874) | “On the balanced one, using big colors because I'm color blind. If it said something more like “left, right” type deal, it would be better.” |
| #29 age 64 (line 987) | “I didn't like the font. I don't know why it bothered me so much” |

| Childish appeal |  |
| --- | --- |
| #10 age 75 (line 387-389) | “I don't know how well I don't know about the age of people who are doing it, but don't know how appealing mermaids and things like that are generally, but it's probably I mean, it is like you see traditional video games, but maybe for younger people but I mean it was adequate.” |
| #13 age 73 (line 504) | “I thought it was kind of juvenile.” |
| #13 age 73 (line 506-508) | “yes, the looks of it were more childish. And I think that you would take it more seriously if it was geared more to our age not that it has to be, you know, like some kind of dangerous thing, but just something that is a little more interesting for an adult.” |
| #26 age 74 (line 928) | “I think it needs to be more age appropriate” |
| #29 age 64 (line 987-988) | I think overall it just felt too childish, you know?” |
| #36 age 69 (line 1137-1138) | “More realistic characters - I haven't seen a mermaid very, very long time, and the circus clown too. Yeah, it could be a little bit more real.” |

| Written feedback interrupted flow |  |
| --- | --- |
| #3 age 62, (line 65—67) | “You focus on two things. You focus on doing the exercise, but you're also looking at how much longer you have left to do it, and if you're struggling with it, you're more desperate to be done with it sooner than it is.” |
| #4 age 57 (line 131-133) | “I prefer it talking to me, rather than the writing, because if I was doing the exercise and I knew I was doing it for so many minutes and I was supposed to be doing it I would have to stop the exercise to read the feedback whereas if I was doing it, and it talked to me. It didn't interrupt my flow.” |
| #35 age 72 (line 1098-1099) | “Other than the messages. You know, your eyes are focused on the character and character perform a function, and when the signs come up, you got to take your eyes off the game to read it.” |

**Question #3: What changes you would like to see as you perform the exercises in the app?**

| Sound feedback |  |
| --- | --- |
| #2 age 71 (line 37) | Maybe, maybe some sound feedback? |
| #4 age 57 (?) (line 131-133) | “I prefer it talking to me, rather than the writing, because if I was doing the exercise and I knew I was doing it for so many minutes and I was supposed to be doing it I would have to stop the exercise to read the feedback whereas if I was doing it, and it talked to me. It didn't interrupt my flow.” |
| #4 age 57(line 131-133) | L: a little bit more maybe feedback saying you have a second left or you have two seconds left or something like that.  Pt: “Yeah, Or your times up rather than it just ending.” |
| #38 age 64 (line 1164-1165) | “I didn't hear any sound. You know, some kind of music or something would be kind of nice. Sounds sound would be good.” |

| Game variation |  |
| --- | --- |
| #6 age 63 (line 215-216) | “maybe change the types of games or what you're doing you know, they're all grabbing the coins or color the lines or maybe you could do some other variations on that thing that so” |
| #14 age 67 (line 562-564, 566) | “It would be kind of a character form of yourself. But yeah, it's some kind of filter. Like say instead of an Ariel they would put the baseball on there or they could, they could make a silly little person moving or something like. To get it so that for all the kind of interest of people and that way they would stick to it.” |
| #38 age 64 (line 1166) | “personalize the character” |

| Increased sensor control/calibration |  |
| --- | --- |
| #1 age 67 (line 13-14) | “It just takes a minute. Sometimes it seems like it got stuck, like when I was doing that this all over. Yeah, the calibration. Just a hair” |
| #8 age 75 (line 291-295) | “Just a little more sensor control. Maybe on the belt is where it’s supposed to be, but I guess that last one just wasn’t controlled. Didn’t seem to be what it should be.” |
| #8 age 75 (line 291-294) | “I would try to move faster and it would tell me I was being abrupt and the timing it seemed to be off at times. Whether it was me from not being used to the doing it or if the sensor wasn’t quite right. Because I would move my head, the app and sensor didn’t always seem to be in sync” |
| #26 age 74 (line 933-934) | “The only thing I saw was the slowness of the tracker” |
| #29 age 64 (line 1006-1007) | it was really fast on one side, but then the other side, you had to go like way over to get it to move. I don't know if that has something to do with the app or what? |

| Speed |  |
| --- | --- |
| #8 age 75 (line 288, 294-295) | “The speed sometimes was off. And then sometimes it seemed for a beginner’s level, it was moving a little too fast” |
| #8 age 75 (line 300-302, 304-305) | “Oh the pause. Because I was trying to keep moving and I could not read them while I was still moving and then it would be up there while I was doing something wrong while I had to move the other direction and then it would disappear. Yeah and I never had a chance to read them while I was moving. It should have paused. Yeah that’s really it- just the speed” |
| #9 age 73 (line 346) | “Maybe if some of it wasn't so fast so I could do it better” |

| Visual appearance |  |
| --- | --- |
| #13 age 73 (line 518-519) | “Yes, I'd like to see it a more appealing game for an older adult that would catch their interest and the other seemed like, oh, this is silly.” |
| #15 age 69 (line 620) | “Well, it did require me to wear my glasses to read those smaller letters. But other than that I can’t think of any.” |
| #32 age 68 (line 1061-1062) | It's a little busy – visually - and I think that kind of takes away from what you're really wanting the patient, or the subject, to do. |

| Instructions (underlined) |  |
| --- | --- |
| #16 age 60 (line 668-669) | “A back arrow” “on any screen like if you go forward and then went well wait a minute. You know, just like on any computer you know you can always back up” |
| #16 age 60 (line 670-674) | “the clown for the balance- better direction there. And then telling you which foot to go on during the balance”  Pt:” and everything else was like you had to make something move to get the coins and this one was It just to me it needed a little bit more explanation.” |
| #32 age 68 (line 1061-1062) | “I would work to make it a little bit more clear. It's a little busy – visually - and I think that kind of takes away from what you're really wanting the patient, or the subject, to do.” |
| #33 age 63 (line 1084-1086) | “If the exercises were harder, the person figure that was on there, they do show you how to do it, but I mean it could be a real person illustration” |
| #35 age 72 (line 1104-1107) | “When doing the balance game, I noticed that for a short point in time, which seemed like an eternity, it seemed like no coins were going out when I was on one foot. And then you'd see the board light up, and then when I went up, the coins started getting real. Couldn't balance myself that well. I just wondered - maybe you should have told me what I was doing.” |

**Question #4: Are there any barriers that would prevent you from using the VestRx at home?**

| **Equipment** |  |
| --- | --- |
| #4 Age 57 (line 148) | “I would not have a stand” |
| #6 Age 63 (line 224) | “Yeah, I guess the only barrier is to always have a stand” |
| #12 Age 70 (line 475) | “well, the equipment, the hardware. I don't have that particular hardware unless you can do it on your cell phone” |
| #11 Age 74 (Line 433) | “The tablet, not everybody has a tablet” |
| #14 Age 67 (line 600-603) | “This has to be transferred over to your cell phone. Because I don't know if there many people use tablet like they used to. So, but the tablets bigger, it's easier to see and stuff. The cell phone would be more portable when you're out and if you're doing these exercises to get rid of the dizziness and you're and you're out someplace, you're going to go to someplace to hide out, to go do” |
| #19 Age 69 (line 791) | “If it would work on your phone, I wouldn’t see this being easy to use on your phone. If you have a desktop at home, would it work on that?” |
| #28 Age 70 (line 966) | “The biggest barrier is the physical pad.” |
| #36 Age 69 (line 1152) | “I would say that I would be concerned for some people. I mean, most people have a phone, but not everybody has a tablet.” |
| #40 Age 69 (line 1206) | “the barriers would be to make sure my device and batteries are charged” |

| **Cost** |  |
| --- | --- |
| #19 Age 69 (line 790) | “Cost” |
| #19 Age 69 (line 768) | “I am concerned about cost of the tablet, the cost of the stand – the cost of my little flashcards was zero.” |

| **Instructions** |  |
| --- | --- |
| #15 Age 69 (line 642) | “So if you're working with people my age, you're going to have to instruct them on how to do that” |
| #22 Age 61 (line 840-843) | “Only for people who aren't computer savvy. My mother, for example, would never be able to figure it out. She's 89, probably wouldn't ask her to, right. My friend's mother would figure it out. So I think just anybody who doesn't know how to use the app or how people can be kind of afraid they're gonna break it.” |
| #32 Age 68 (Line 1064) | “When we first did it, it was a little confusing, so yes, I do. If I were to buy it from the shelf and take it home, that wouldn't be the way to do that. You need a tutorial with it.” |

| **Home/Setup** |  |
| --- | --- |
| #11 Age 74 (line 428-430) | “It did say that you had to try and mount the thing at eye level when you're standing up that might be a little difficult. I don't know. You probably would have to improvise something like this or put it on a shelf or whatever, yeah.” |
| #13 Age 73 (line 523) | “Well, I I'd have some corners, I think maybe the level you know of where to put it, people would have kind of figure that out to get it up higher” |
| #18 Age 74 (line 748-751) | “OK, the only thing it probably needs to be clear at as to like to your point about where you set it up or how you set it up would make a difference because it's one thing to watch a spot on the wall and do your exercises. That’s easy. But the only thing I wear bifocals, so it's hard to figure out whether if I put it up against the wall I can stand back and look through the top part of my glasses.” |
| #19 Age 69 (line 790) | “Where you would put the tablet. How easy it would be to see” |
| #19 Age 69 (line 790) | “I am concerned with the ones where you are leaning to the back and the side- people in their house may not have the space where they can easily do it. When I would do my work with these little cards, I would put my cards above on the window and I would hold on to the kitchen sink and that way if I leaned back, I could grab there. And some of the most useful exercises were done with my eyes closed and so this wouldn’t work there.” |
| #23 Age (Line 862) | “Just having it set up in my home.” |
| #24 Age 60 (line | “As long as you have a chair or something, set up is going to be fine” |
| #25 Age 61 (line 910) | “I couldn’t do that. I couldn’t do it because I would have to have my son and my daughter do that kind of stuff. Because, like I said, I got a flip phone in my pocket.” |
| #28 Age 70 (line 968) | “I think if I'm going to do this at home, the only thing I have to figure out is how I would put the tablet up. Although there's another way to do that - I could do it by using Chromecast to a television! So, enabling Chromecast on the app. That would be good. Then I can see how I can do it.” |
| #29 Age 64 (line 1010) | “But I think the idea of hooking it up to like a computer screen or a TV screen - I mean I don't have a TV - but yeah, computer screen - so it's a little bit bigger.” |
| #15 Age 69 (line 1119) | “Anything you do with the computer, you got to set it up. I think it'd be an asset to a person - to a patient.” |

| **Time** |  |
| --- | --- |
| #35 Age 72 (line 1117) | “No, like anything else, it’s about finding time. But I mean, if you're serious about it, if you know you need it, you're going to.” |
| #26 Age 74 (line 936) | “Just like with a sheet of exercises, you either do them, or you don’t.” |
